# Norwegian general practitioners’ and radiologists’ perspectives on the referral, justification, and unnecessary imaging—a survey

**DOI:** 10.1080/02813432.2024.2366247

**Published:** 2024-06-25

**Authors:** Elin Kjelle, Eivind Richter Andersen, Ingrid Øfsti Brandsæter, Bjørn Morten Hofmann

**Affiliations:** a Institute for the Health Sciences at the Norwegian University of Science and Technology (NTNU) at Gjøvik, Gjøvik, Norway; b Centre of Medical Ethics, Centre of Medical Ethics at the University of Oslo, Blindern, Norway

**Keywords:** General practitioner, referral, diagnostic imaging, justification, radiologist, Overutilization, Survey

## Abstract

**Aim:**

This study aimed to survey general practitioners’ (GPs) and radiologists’ perspectives on referrals, imaging justification, and unnecessary imaging in Norway.

**Materials and methods:**

The survey covered access to imaging, responsibilities, attitudes toward justification assessment, referral process, and demographics using multiple choice questions, statements to report agreement with using the Likert scale and one open question.

**Results:**

Forty radiologists and 58 GPs attending national conferences completed a web-based survey, with a 20/15% response rate, respectively. Both radiologists (97%) and GPs (100%) considered avoiding unnecessary examinations essential to their role in the healthcare service. Still, 91% of GPs admitted that they referred to imaging they thought was not helpful, while about 60% of the radiologists agreed that unnecessary imaging was conducted in their workplace. GPs reported pressure from patients and patients having private insurance as the most common reasons for doing unnecessary examinations. In contrast, radiologists reported a lack of clinical information and the inability to discuss patient cases with the GPs as the most common reasons.

**Conclusion:**

This study adds to our understanding of radiologists’ and GPs’ perspectives on unnecessary imaging and referrals. Better guidelines and, even more importantly, better communication between the referrer and the radiologist are needed. Addressing these issues can reduce unnecessary imaging and improve the quality and safety of care.

## Introduction

Diagnostic imaging is a cornerstone in modern medicine, and with continuous improvement of diagnostic efficacy and increased access, imaging has increased worldwide [1]. However, several imaging examinations can be of low value as they do not contribute information relevant to further treatment and care [2,3]. Such tests are unnecessary or inappropriate and may lead to overdiagnosis and overtreatment that can harm patients [4–7]. Further, overutilizing imaging and treatment wastes sparse healthcare resources and causes ineffective health services and significant opportunity costs [1,3].

Several imaging modalities use ionizing radiation, and all diagnostic imaging (including nonionizing examinations) must be justified. The risk of the imaging (e.g. due to radiation exposure) should be lower than the benefit from the examination (sufficient net benefit) [8]. According to the international safety standard from the International Atomic Energy Agency (IAEA) - Radiation Protection of Patients (RPOP), the referrer and the radiologist should, in consultation, carry out the individual justification for patients [9]. This principle is materialized in radiation protection legislation internationally and in Norway [10].

Still, general practitioners (GPs) may refer patients for unnecessary examinations, and radiologists may choose to accept these referrals. There can be many reasons for such adverse practices, such as high expectations, system demands of production volume, overconfidence in imaging among patients and physicians, or to please the patient’s expectations [11,12].

In Norway, radiologists work in hospitals (public or nonprofit) or semi-private imaging centers. GPs work in small primary care offices or municipal emergency rooms. The primary communication between the referrer and the radiologist is through digital referrals. A referral of good quality is essential for the radiologist to contribute to the justification process [13]. Alternative forms of communication have been reported to be difficult, especially between radiologists and GPs or radiologists and consultants in private practice [14].

Variable referral quality often results in more inappropriate imaging and poor quality of imaging results and subsequent diagnostics [15]. Unnecessary imaging is a well-known problem [1–3], despite the development of several guidelines, recommendations, and interventions developed to support referrers and reduce unnecessary imaging [16–20].

In Norway, justification is a joint responsibility between the referrer and radiologist, where a radiologist should assess all referrals according to the radiation protection legislation [10]. In a study involving 480 Norwegian radiologists, 76% reported receiving referrals lacking information during their most recent workday, and 63% reported they sometimes disagreed with referrers that imaging was indicated [21]. Still, only 1% of referrals to CT and MRI examinations are rejected due to lack of medical indication [21]. Building on a previous survey among radiologists [22], this study explored referral practice and attitudes toward unnecessary imaging by including the GPs’ perspective. Thus, the objective was to survey radiologists’ and GPs’ perspectives on referrals, imaging justification, and unnecessary imaging in Norway. As such, the study is unique in presenting the perspectives of two key stakeholders in imaging.

## Materials and methods

Two questionnaires, one for radiologists and one for GPs, were developed. All authors were involved in the development of the questionnaires. The questionnaires started with an information section informing participants of the study’s objective, data handling and storing, and plans for publication. Further, the questions covered demographics, access to imaging, responsibility, attitudes toward justification assessment, referral process, and reasons for doing or avoiding unnecessary imaging. Questions were designed as multiple-choice questions (1–3 possible ticks), statements with a four-point Likert scale with ‘Strongly agree,’ ‘Agree,’ ‘Disagree,’ and ‘Strongly disagree’ including an option of ‘Don’t know,’ and one open question (‘Describe what you characterize as a justified/appropriate imaging examination’).

The questionnaires were designed online in Norwegian, using ‘Nettskjema’ (https://nettskjema.no/). The questions were based on a review of existing literature and a previous study on radiologists’ perceptions of the causes of the increasing and unnecessary use of diagnostic imaging in Norway [22]. Three radiologists and two GPs piloted the questionnaire. Based on their feedback, amendments were made to align with their understanding of questions and concepts and to provide relevant alternatives. The questionnaires are available in additional files 1 and 2.

### Data collection and analysis

All authors contributed to both data collection and analysis. Data were collected in October 2022 during two separate conferences in Oslo, gathering GPs and radiologists, respectively, from across the country. The participants were invited to answer the questionnaire during two and three conference days for GPs and radiologists, respectively. Participants were invited through a research group member, contacting them personally at the venue. This strategy was chosen as busy physicians often ignore survey invitations *via* mail, e-mail, or social media. In addition, radiologists and GPs’ email addresses are not readily available for survey distribution through any open channels or organization. Moreover, these conferences offer courses necessary for maintaining physician’s specialization and thus recruit various participants from different parts of the country. Volunteers gained access to the questionnaire by scanning a QR code on their mobile phones or using available computers to access the form. Residents in radiology and general medicine and physicians in training in these fields were eligible to participate in the survey. Due to the moderate number of participants, data were analyzed through descriptive statistics using Microsoft^®^ Excel^®^ for Microsoft 365 MSO (Version 2207). Free text responses were analyzed using quantitative content analysis, sorting similar statements into categories and quantifying the number of statements in each category.

### Ethics

All participants volunteered and gave written consent to participate in the survey and the scientific publication of the results. Data entered in the questionnaire were anonymous. This survey did not require approval from a research ethics committee, as no personal information was gathered.

## Results

The survey received 98 responses: 40 from radiologists and 58 from GPs working in Norway; this was 22% and 15% of the preregistered attendants of the radiologist (*n* = 180) and GP conference (*n* = 398), respectively. The demographics of the participants are presented in Table 1.

**Table 1.** Demographics of the participants.

Radiologist - *n* (%)	General practitioner (GP) - *n* (%)
Years of experience	
<5 years − 5 (12) 5–10 years − 9 (23) >10 years − 26 (65)	<5 years − 18 (31) 5–10 years − 17 (29) >10 years − 23 (40)
Specialist in radiology/general medicine	
Yes − 34 (85) No − 6 (15)	Yes − 32 (55) No − 26 (45)
Place of work	
Local/Nonprofit hospital − 18 (45) University Hospital − 22 (55)	GPs office − 50 (86) GPs office and emergency room − 5 (9) Other − 3 (5)
Subspecialty (>1 subspeciality possible)	
General radiology − 19 Neuroradiology − 7 Chest − 8 Abdominal − 6 Musculoskeletal − 4 Cardiac/vascular − 3 Breast − 3 Pediatric − 3 Interventional − 4	

Among the participants, 70% of radiologists and 24% of GPs considered Norway’s imaging capacity too low. Further, when GPs were asked whether they sometimes refer to examinations with doubtful value, 91% answered yes.

### Justification: responsibility and attitude

In the first question, the respondents should describe their understanding of a justified examination in free text. Only 10% of radiologists and 3.5% of GPs described a justified examination as imaging with sufficient net benefit, as defined in the radiation protection recommendations and regulations. Most radiologists (77%) and GPs (57%) described justified imaging as examinations with a therapeutic consequence. 31% of GPs described a justified examination as imaging with a clinical indication. No radiologist used this description. 12% of radiologists and 5% of GPs wrote that an examination is justified if sufficient information is given in the referral. The remaining answers were blank or had other descriptions.

When asked to consider statements on justification, 78% (80% of the radiologists and 76% of the GPs) agreed that the justification of the examinations is a joint responsibility between GPs and radiologists. Further, 18% of the radiologists and 2% of GPs considered justification a radiologist’s responsibility, and about 3% of the radiologists and 22% of the GPs considered the GP responsible for justification of examinations.

**Figure 1.**
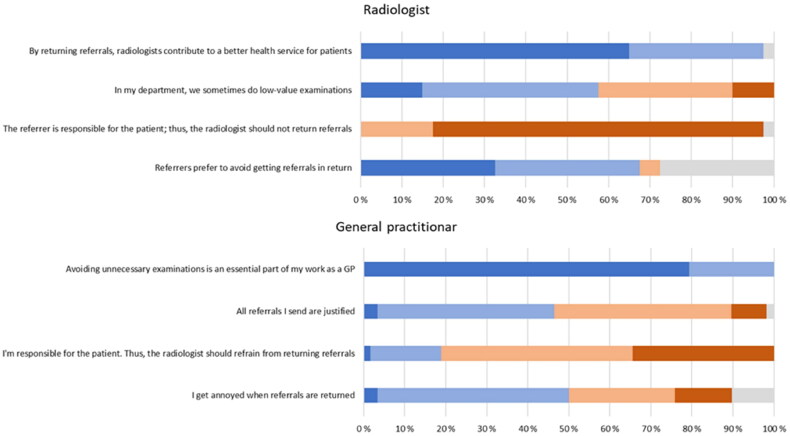
Overview of the radiologists’ and GPs’ agreement rate to statements on justification. Color code: Dark blue represents ‘strongly agree,’ light blue represents ‘agree,’ light orange represents ‘disagree,’ and dark orange represents ‘strongly disagree.’ grey represents ‘do not know.’

Figure 1 shows details of statements the respondents were asked to consider. When asked whether avoiding unnecessary examinations was essential to their role and whether this contributes to better healthcare quality, 97% of radiologists and all GPs agreed or strongly agreed. At the same time, almost 60% of radiologists agreed or strongly agreed that unnecessary examinations were conducted in their department. Among the GPs, only 47% of the referrers agreed or strongly agreed that all their referrals were justified.

Ninety-seven percent of radiologists and 81% of GPs disagreed or strongly disagreed with the statement, ‘The referrer is/I am responsible for the patient; thus, radiologists should not be able to reject referrals.’ At the same time, 50% of GPs agreed or strongly agreed that they were annoyed when referrals were rejected. Among the radiologists, 69% agreed or strongly agreed that referrers would like to avoid referral rejection. This statement had the most ‘do not know’ answers in the survey (28%).

### The referral processes

**Figure 2.**
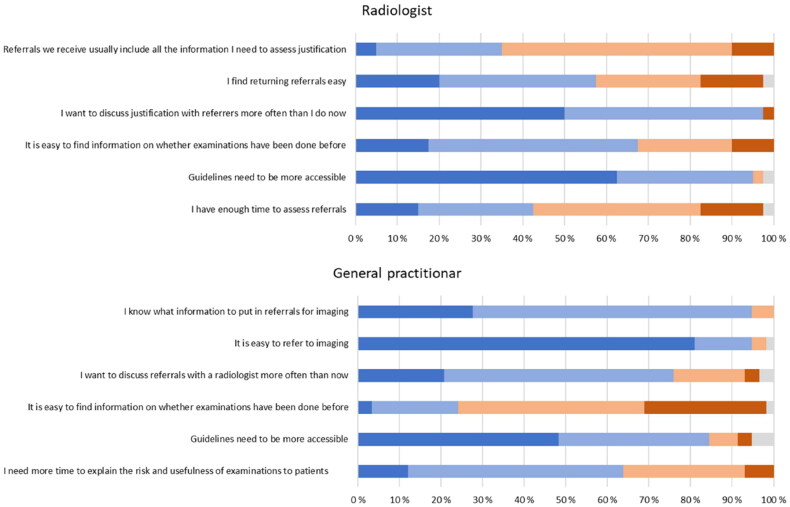
Overview of the radiologists and GPs’ agreement rate to statements on the referral process. Color code: Dark blue represents ‘strongly agree,’ light blue represents ‘agree,’ light orange represents ‘disagree,’ and dark orange represents ‘strongly disagree,’ grey represents ‘do not know.’

In statements related to the referral process (Figure 2), virtually all the GPs (95%) agreed or strongly agreed that they know what information to put in a referral. In comparison, only 33% of radiologists agreed or strongly agreed that referrals include all required information for justification assessment. About 95% of GPs agreed or strongly agreed that it is easy to refer to imaging, while nearly 60% of radiologists agreed or strongly agreed that rejecting referrals is easy. More than 97% of the radiologists and almost 76% of the GPs agreed or strongly agreed that they would like to discuss referrals and justification with each other more often. More radiologists (69%) than GPs (24%) agreed or strongly agreed that it is easy to find information on whether an examination has already been done. Further, 95% of the radiologists and 85% of the GPs agreed or strongly agreed that imaging guidelines should be more accessible.

Fifty-six percent of radiologists agreed or strongly agreed they needed more time to assess referrals. In comparison, 65% of GPs agreed or strongly agreed that they needed more time to explain the risk and necessity of imaging to patients.

### Unnecessary imaging

When asked what causes unnecessary imaging, pressure from patients or referrers was the most common reason. 83% of GPs felt pressured by patients, and 54% of radiologists felt pressured by referrers to do imaging that might be unnecessary.

**Figure 3.**
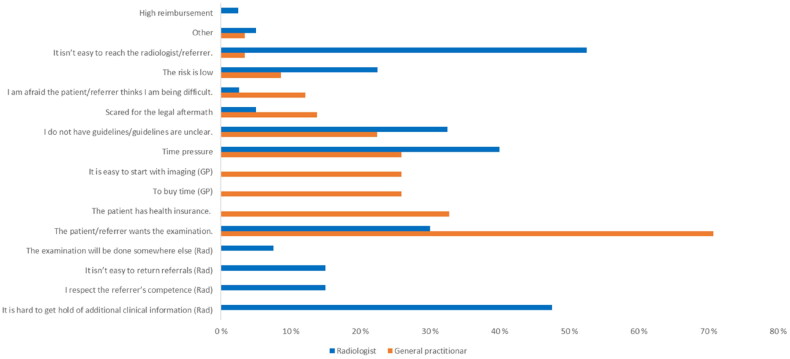
GPs And radiologists’ reasons for referring to or approving examinations of doubtful usefulness. Reasons marked with (GP) are for GPs only, and those marked with (rad) are reasons available to radiologists only.

In a list of possible reasons (13 for radiologists and 18 for GPs), respondents could choose up to three causes for using unnecessary imaging in their clinical practice. The most common (>30%) reasons for the GPs were: ‘The patient wants the examination’ (71%) and ‘The patients have health insurance’ (33%). Among the radiologists, reasons given for accepting referrals with uncertain benefits were most commonly (>30%): ‘It is hard to reach the referrer’ (54%), ‘It is hard to get additional clinical information’ (49%), ‘Time pressure’ (41%), ‘I do not have any guidelines or guidelines are unclear’ (33%), ‘The patient/referrer wants the examination’ (31%). These results and further details are presented in Figure 3.

No GPs chose reimbursement or efficiency demands as a reason for referring to unnecessary imaging, and none of the radiologists chose reasons related to health insurance, efficiency demands, or the rejection of referrals’ lack of effect on the quality of health service provided.

### When unnecessary imaging is avoided

**Figure 4.**
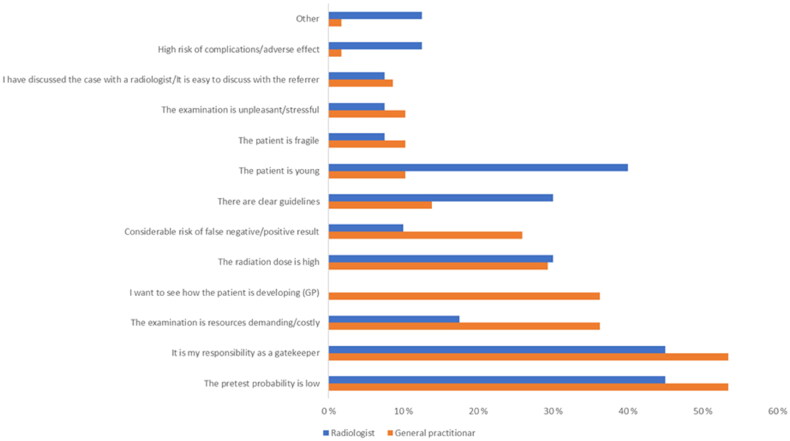
GPs And radiologists’ reasons for avoiding/rejecting examinations of doubtful usefulness. (GP)=reason was given just for GPs.

Radiologists and GPs could choose up to three alternatives from a list of 12 (for radiologists) or 13 (for GPs) statements for reasons to avoid unnecessary imaging in their clinical practice. The most frequently answered statements among GPs (53%) and radiologists (46%) were ‘It is my responsibility as a gatekeeper’ and ‘The pretest probability is low.’ Further, the GPs chose reasons as follows: ‘I want to see how the patient is developing’ (36%), ‘The radiation dose is high’ (29%), or ‘Risk of false negative/positive results’ (26%). The radiologists’ most common reasons for rejecting referrals were: ‘The patient is young’ (41%), ‘The radiation dose is high’ (31%), or ‘There are clear guidelines’ (28%). These results and further details are presented in Figure 4.

## Discussion

This study identified that most radiologists and GPs had a similar understanding of the responsibilities for avoiding unnecessary imaging and assessing justification. Almost all respondents considered avoiding unnecessary imaging a vital part of their role and essential for a high-quality health service. Still, more than half of the respondents admitted to referring to or approving unnecessary examinations. These findings align with earlier research that has reported that radiologists find prioritizing difficult [14], and for GPs, the gatekeeper function is challenging to uphold because of increased pressure from patients [23].

Imaging capacity in Norway and other Western countries is under pressure. While the number of examinations is steadily increasing, there is a lack of radiologists, especially in smaller local or rural hospitals [1,3,21]. Our study indicates that GPs considered imaging capacity adequate, while most radiologists considered imaging capacity low. One possible explanation for this discrepancy could be that the GPs perceive easier access to imaging services within their general practice, which private imaging centers often serve. These centers typically offer shorter wait times than hospitals [24]. The radiologists, on the other hand, may adopt a broader perspective, taking into account outpatients and inpatients, along with the prolonged wait time characteristics of the public healthcare setting [24].

When asked to explain a justified examination briefly, most respondents wrote “imaging with a therapeutic consequence” or “clinically indicated.” Only a few radiologists and GPs considered a risk as part of the justification assessment. Thus, there seems to be a lack of knowledge on the principle of justification. According to Norwegian legislation, all health personnel shall conduct their work in accordance with the requirements of professional responsibility and diligent care that can be expected based on their qualifications, the nature of their work, and the situation in general, and avoid unnecessary costs and use of resources [25]. Further, GPs and radiologists are obligated only to refer or allow imaging that is justified and in accordance with guidelines or best practices [10], and all radiation exposure should be kept to a practical minimum [8]. However, there still seem to be barriers for GPs and radiologists to comply with these obligations in clinical practice.

Time pressure and pressure from patients were reported as prominent reasons for unnecessary imaging by the GPs in the present study, where lack of time to describe and discuss the risks of imaging with patients was underscored. This is supported by earlier research, showing how GPs are pressured through system processes [12,14,23,26,27]. However, patients’ overusing services can be a reason for increased time pressure on GPs and radiologists. Thus, reducing overuse might need some effort now but reduce pressure later. Both radiologists and GPs agreed that imaging guidelines should be more readily available. In Norway, GPs have access to national guidelines. However, few have them available directly in the electronic referral system. Electronic guidelines in the referral system are common in other European countries [28]. Moreover, guidelines can be helpful but can also drive unnecessary imaging. They should, therefore, be used with careful consideration [14,20].

Almost all GPs found it easy to refer to imaging, and about half of the GPs answered that they would be annoyed if a referral was rejected; this suggests reduced latitude for radiologists to enforce justification assessment. Specifically, almost half of the radiologists found it challenging to reject referrals. The discrepancies between the ease of referring to imaging and the challenges in rejecting referrals may be an essential driver for unnecessary imaging [26].

Almost all radiologists wanted better communication with GPs, and a vast majority of the GPs agreed that contacting a radiologist would be helpful in the referral process. Poor-quality referrals are considered a critical problem in diagnostic imaging [21,29], and radiologists in this study reported a lack of access to clinical information about patients. The GPs reported a lack of information on imaging history. Facilitating communication between these two professional groups could help reduce the use of unnecessary imaging [14,26]. Thus, better communication lines or access to information across health service levels could improve this situation. Thus, there seems to be a need to find a well-functioning solution to facilitate better use of guidelines and cooperation between GPs and radiologists.

### Strengths and limitations

As with all surveys, we strive for high response rates to obtain representability. However, if representability can be demonstrated [30], surveys will also be valid with lower response rates, as designated studies show [31,32]. Moreover, the response rates align with earlier research [33], and our results from questions similar to previous surveys fit well with other studies from a Norwegian setting [14,22]. However, those who answer questionnaires may be more active in the societies or more interested in professional development than the average GP or radiologist. Thus, the findings in this study are not necessarily representative of all GPs and radiologists or transferable to other settings.

In this study, we only included GPs as referring clinicians. By including in-house clinicians from hospitals, other relevant issues could be revealed. However, patient assessment can be challenging for GPs, especially at the start of the diagnostic process, where the value of imaging may be unclear [14]. Hence, GPs are a warranted target group when investigating low-value practices. Despite its limitations, this descriptive study gives an insight into radiologists’ and GPs’ perspectives on unnecessary imaging and referral practices in Norway.

In conclusion, the respondents in this survey indicated that both GPs and radiologists find avoiding unnecessary imaging an essential but challenging part of their tasks. They admit to referring to or performing examinations of low value in defiance of regulations. This threatens to undermine the safety, quality, and efficiency of care. To reduce low-value imaging utilization, higher referral quality and better communication between GPs and radiologists are needed in the justification assessment. Further, both radiologists and GPs called for better and more accessible guidelines. More research is required to provide a more comprehensive overview of radiologists’ and GPs’ perspectives on these issues.

## Supplementary Material

Supplemental Material

Supplemental Material

## Data Availability

The datasets used and/or analyzed during the current study are available from the corresponding author upon reasonable request.

## Additional files

Additional file 1 –The questionnaire used for radiologists.

Additional file 2 –The questionnaire used for GPs.
