# Supplementary material for: Norwegian general practitioners’ and radiologists’ perspectives on the referral, justification, and unnecessary imaging—a survey
Source: Scand J Prim Health Care. 2024 Jun 25;42(4):574–81. doi: 10.1080/02813432.2024.2366247 (PMC11552244; doi:10.1080/02813432.2024.2366247)
Supplement: Supplemental Material [file IPRI_A_2366247_SM0316.docx]

**Berettigelse av bildediagnostiske undersøkelser**

**Berettigelse av bildediagnostiske undersøkelser**

Dette er en invitasjon til å delta i en spørreundersøkelse som omhandler indikasjon for og bruk av bildediagnostiske undersøkelser.

**Formål**

Målet med undersøkelsen er å kartlegge holdninger og rutiner for vurdering av berettigelse og bruken av lavverdi radiologiske tjenester, slik at tjenestene kan forbedres. Resultatene fra undersøkelsen vil bli publisert i vitenskapelige tidsskrift.

**Hvem er ansvarlig for forskningsprosjektet?**

xxxxxx er ansvarlig for prosjektet "xxxxx" som denne spørreundersøkelse er en del av*.*

**Hvorfor får du spørsmål om å delta?**

Vi spør deg om å delta fordi du som radiolog/nukleærmedisiner eller lege i spesialisering sitter på viktig erfaring og kunnskap om henvisning til radiologiske tjenester.

**Hva innebærer det for deg å delta?**

Hvis du velger å delta i prosjektet, innebærer det at du fyller ut et spørreskjema. Det vil ta deg ca. 5 minutter. Spørreskjemaet inneholder spørsmål vedrørende holdninger til og rutiner for henvisning til radiologiske tjenester og vurdering av berettigelse. Dine svar fra spørreskjemaet blir registrert elektronisk.

**Det er frivillig å delta**

Det er frivillig å delta i spørreundersøkelsen og prosjektet lagrer ingen personidentifiserende data om deg.

**Ditt personvern**

Vi vil bare bruke opplysningene til formålene vi har fortalt om i dette skrivet. Vi behandler opplysningene konfidensielt og anonymisert. Vi ber om at du ikke skriver noe i fritekstfeltene som kan identifisere deg eller andre personer.

**Hva skjer med personopplysningene dine når forskningsprosjektet avsluttes?**

Prosjektet vil etter planen avsluttes 31.12.2023. Etter prosjektslutt vil alt datamaterialet slettes.

Hvis du har spørsmål til prosjektet, ta kontakt med:

*xxxxxx*

**Ved å sende inn dine svar på denne undersøkelsen gir du ditt samtykke til å delta.**

**Start her!**

**Beskriv hva du mener kjennetegner en berettiget/indisert undersøkelse.**

**Hvem er ansvarlig for at en radiologisk/nukleærmedisinsk undersøkelse er**

**berettiget?**

Radiolog/Nukleærmedisiner

Henviser

Begge

**Under ser du noen påstander om vurdering av henvisninger fra eksterne henvisere (f.eks fastlege)**

Ta stilling til hvor enig eller uenig du er i påstandene.

**Henvisningene vi får tilsendt, inneholder som regel all informasjon jeg trenger for å vurdere**

**berettigelse**

Helt enig

Litt enig

Litt uenig

Helt uenig

Vet ikke

**Jeg ønsker mer kontakt med henviser ved spørsmål om berettigelse**

Helt enig

Litt enig

Litt uenig

Helt uenig

Vet ikke

**Det er enkelt å finne ut av om undersøkelsen er gjennomført tidligere**

Helt enig

Litt enig

Litt uenig

Helt uenig

Vet ikke

**Henvisere liker ikke å få henvisninger returnert**

Helt enig

Litt enig

Litt uenig

Helt uenig

Vet ikke

**Jeg synes det er enkelt å returnere henvisninger**

Helt enig

Litt enig

Litt uenig

Helt uenig

Vet ikke

**Henviser er ansvarlig for pasienten derfor skal ikke radiologer avvise undersøkelser**

Helt enig

Litt enig

Litt uenig

Helt uenig

Vet ikke

**Hos oss gjøres undersøkelser selv om de er til liten nytte for pasientens videre utredning og**

**behandling**

Helt enig

Litt enig

Litt uenig

Helt uenig

Vet ikke

**Ved å returnere henvisninger bidrar radiologer/nukl.med til en bedre helsetjeneste for**

**pasientene**

Helt enig

Litt enig

Litt uenig

Helt uenig

Vet ikke

**Henviser presser meg ofte til å gjennomføre unødvendige undersøkelser**

Helt enig

Litt enig

Litt uenig

Helt uenig

Vet ikke

**Retningslinjer må være mer tilgjengelige**

Helt enig

Litt enig

Litt uenig

Helt uenig

Vet ikke

**Jeg har nok tid til å vurdere henvisninger**

Helt enig

Litt enig

Litt uenig

Helt uenig

Vet ikke

**Du betviler nytten av en undersøkelse henvist fra primærhelsetjenesten, kryss av**

**for de vanligste årsakene til at undersøkelsen likevel utføres**

Du kan maksimalt velge 3 av årsakene på listen.

Pasienten/henviser ønsker undersøkelsen

Jeg mangler gode retningslinjer/retningslinjene er uklare

Tidspress

Det er vanskelig å få tak i henviser

Høy refusjon for undersøkelsen

Stor faglig respekt for henviserens vurdering

Det er vanskelig å innhente tilleggsinformasjon

Frykt for rettslig etterspill

Det er et produktivitetskrav på arbeidsplassen

Det er tungvint å returnere henvisninger

Er redd for å bli sett på som vanskelig

Risiko for pasienten er lav

Pasienten har helseforsikring

Å avvise undersøkelser uten klar indikasjon bidrar ikke til en mer effektiv helsetjeneste

Undersøkelser vil uansett bli tatt hos en annen aktør

Annet, spesifiser:

**Spesifiser her:**

*Dette elementet vises kun dersom alternativet «Annet, spesifiser:» er valgt i spørsmålet «Du betviler nytten av en undersøkelse henvist fra*

*primærhelsetjenesten, kryss av for de vanligste årsakene til at undersøkelsen likevel utføres»*

**Du betviler nytten av en undersøkelse henvist eksternt. Kryss av for de vanligste**

**årsakene til at henvisningen avvises**

Du kan maksimalt velge 3 av årsakene på listen.

Pasienten er ung (barn, ungdom)

Pasienten er skrøpelig

Undersøkelsen er ubehaglig eller belastende for pasienten

Stor risiko for alvorlige komplikasjoner eller bivirkninger

Undersøkelsen er ressurskrevende (tid eller penger)

Pretestsannsynlighet er lav

Det er enkelt å ha dialog med henviser og bli enige om retur

Det finnes klare retningslinjer

Det er mitt ansvar som radiolog

Høy stråledose ved undersøkelsen

Stor risiko for falske positive/negative funn

Annet, spesifiser:

**Spesifiser her:**

*Dette elementet vises kun dersom alternativet «Annet, spesifiser:» er valgt i spørsmålet «Du betviler nytten av en undersøkelse henvist eksternt.*

*Kryss av for de vanligste årsakene til at henvisningen avvises»*

**Bakgrunnsinformasjon**

**Er du godkjent spesialist i radiologi/nukleærmedisin?**

Ja

Nei

**I hvor mange år har du arbeidet innen radiologi/nukl?**

Inkluderer både arbeid som LIS/assistentlege og spesialist.

&lt;5år

5-10år

&gt;10år

**Hvordan er kapasiteten på det bildediagnostisike tilbudet innen ditt fagområde på**

**ditt arbeidssted?**

Det er ledig kapasitet til å ta imot flere pasienter

Det er tilstrekkelig kapasitet i forhold til pasientgrunnlaget

Det er mangelfull kapasitet med lange ventetider for pasientene

**Ved hvilken type institusjon har du ditt daglige arbeid?**

Stort offentlig sykehus (universitetssykehus eller tidligere regions-/sentralsykehus)

Mindre offentlig sykehus (tidligere lokal-/fylkessykehus)

Røntgeninstitutt

Annet, spesifiser:

**Spesifiser her:**

*Dette elementet vises kun dersom alternativet «Annet, spesifiser:» er valgt i spørsmålet «Ved hvilken type institusjon har du ditt daglige arbeid?»*

**Subspesialitet/arbeidsområde**

Barneradiologi

Nevroradiologi

Thorax

Abdomen/urologi/gyn.

Muskel/skjellett

Hjerte/kar

Brystdiagnostikk

Generell radiologi

Intervensjonsradiologi

Annet, spesifiser:

**Spesifiser her:**

*Dette elementet vises kun dersom alternativet «Annet, spesifiser:» er valgt i spørsmålet «Subspesialitet/arbeidsområde»*

*Generert: 2023-05-31 08:42:39.*
