# Supplementary material for: Norwegian general practitioners’ and radiologists’ perspectives on the referral, justification, and unnecessary imaging—a survey
Source: Scand J Prim Health Care. 2024 Jun 25;42(4):574–81. doi: 10.1080/02813432.2024.2366247 (PMC11552244; doi:10.1080/02813432.2024.2366247)
Supplement: Supplemental Material [file IPRI_A_2366247_SM0315.docx]

**Berettigelse av radiologiske undersøkelser - allmennlege**

**Berettigelse av radiologiske undersøkelser**

Dette er en invitasjon til å delta i en spørreundersøkelse som omhandler indikasjon for og bruk av radiologiske undersøkelser.

**Formål**

Målet med undersøkelsen er å kartlegge holdninger og rutiner for vurdering av berettigelse og bruken

av lavverdi radiologiske tjenester, slik at tjenestene kan forbedres. Resultatene fra undersøkelsen vil bli publisert i vitenskapelige tidsskrift.

**Hvem er ansvarlig for forskningsprosjektet?**

xxxxxx er ansvarlig for prosjektet "xxxxxxxxx" som denne spørreundersøkelse er en del av*.*

**Hvorfor får du spørsmål om å delta?**

Vi spør deg om å delta fordi du som radiolog/allmennpraktiker eller lege i spesialisering sitter på viktig erfaring og kunnskap om henvisning til radiologiske tjenester.

**Hva innebærer det for deg å delta?**

Hvis du velger å delta i prosjektet, innebærer det at du fyller ut et spørreskjema. Det vil ta deg ca. 5 minutter. Spørreskjemaet inneholder spørsmål vedrørende holdninger til og rutiner for henvisning til radiologiske tjenester og vurdering av berettigelse. Dine svar fra spørreskjemaet blir registrert elektronisk.

**Det er frivillig å delta**

Det er frivillig å delta i spørreundersøkelsen og prosjektet lagrer ingen personidentifiserende data om deg.

**Ditt personvern**

Vi vil bare bruke opplysningene til formålene vi har fortalt om i dette skrivet. Vi behandler opplysningene konfidensielt og anonymisert. Vi ber om at du ikke skriver noe i fritekstfeltene som kan identifisere deg eller andre personer.

**Hva skjer med personopplysningene dine når forskningsprosjektet avsluttes?**

Prosjektet vil etter planen avsluttes 31.12.2023. Etter prosjektslutt vil alt datamaterialet slettes.

Hvis du har spørsmål til prosjektet, ta kontakt med:

*xxxxxxxxxx*

**Ved å sende inn dine svar på denne undersøkelsen gir du ditt samtykke til å delta.**

**Start her!**

**Beskriv hva du mener kjennetegner en berettiget/indisert radiologisk undersøkelse.**

**Hvem er ansvarlig for at radiologiske undersøkelser er berettiget?**

Radiolog

Henviser

Begge

**Under ser du noen påstander om henvisning til radiologi**

Ta stilling til hvor enig eller uenig du er i påstandene.

**Det er enkelt å henvise til radiologi**

Helt enig

Litt enig

Litt uenig

Helt uenig

Vet ikke

**Jeg vet hvilken informasjon som er nødvendig i en henvisning til radiologi**

Helt enig

Litt enig

Litt uenig

Helt uenig

Vet ikke

**Jeg ønsker mer kontakt med radiolog i henvisningsprosessen**

Helt enig

Litt enig

Litt uenig

Helt uenig

Vet ikke

**Det er enkelt å finne ut av om undersøkelsen er gjennomført tidligere**

Helt enig

Litt enig

Litt uenig

Helt uenig

Vet ikke

**Jeg er ansvarlig for pasienten, derfor skal ikke radiologer kunne avvise min henvisning**

Helt enig

Litt enig

Litt uenig

Helt uenig

Vet ikke

**Jeg blir irritert når henvisninger kommer i retur**

Helt enig

Litt enig

Litt uenig

Helt uenig

Vet ikke

**Alle undersøkelser jeg henviser til, er nyttige for pasientens videre behandling**

Helt enig

Litt enig

Litt uenig

Helt uenig

Vet ikke

**Å unngå unødvendige undersøkelser er en viktig jobb for meg som allmennlege**

Helt enig

Litt enig

Litt uenig

Helt uenig

Vet ikke

**Pasienter presser meg ofte til å henvise til unødvendige undersøkelser**

Helt enig

Litt enig

Litt uenig

Helt uenig

Vet ikke

**Retningslinjer må være mer tilgjengelige**

Helt enig

Litt enig

Litt uenig

Helt uenig

Vet ikke

**Jeg har for liten tid til å forklare pasienten om nytte og risiko ved en undersøkelse**

Helt enig

Litt enig

Litt uenig

Helt uenig

Vet ikke

**Hender det at du sender henvisning til undersøkelser du betviler nytten av?**

Ja

Nei

**Når du sender henvisninger du er usikker på nytten av, kryss av for de vanligste**

**årsakene til at slike henvisninger sendes**

*Dette elementet vises kun dersom alternativet «Ja» er valgt i spørsmålet «Hender det at du sender henvisning til undersøkelser du betviler nytten*

*av?»*

Du kan maksimalt velge 3 av årsakene på listen.

Pasienten/pårørende ønsker undersøkelsen

Jeg mangler gode retningslinjer/retningslinjene er uklare

Kjøper meg tid når jeg er usikker

Det er vanskelig å få tak i radiolog

Tidspress

Gir høyere refusjon

Radiologi er et enkelt tiltak å starte med

Risiko for pasienten er lav

Redd for å skade forholdet til pasienten

Pasienten har helseforsikring

Det er et produktivitetskrav på arbeidsplassen

Frykt for rettslig etterspill

Annet, spesifiser:

**Spesifiser her:**

*Dette elementet vises kun dersom alternativet «Annet, spesifiser:» er valgt i spørsmålet «Når du sender henvisninger du er usikker på nytten av,*

*kryss av for de vanligste årsakene til at slike henvisninger sendes»*

**Når du lar være å sende henvisninger til undersøkelser du er usikker på om er nyttig for pasienten, hva er de vanligste årsakene til at henvisningen ikke sendes**

Du kan maksimalt velge 3 av årsakene på listen.

Høy stråledose ved undersøkelsen

Pretestsannsynlighet er for lav

Pasienten er ung (barn, ungdom)

Pasienten er skrøpelig

Undersøkelsen er ubehaglig eller belastende for pasienten

Stor risiko for alvorlige komplikasjoner eller bivirkninger

Undersøkelsen er ressurskrevende (tid eller penger)

Jeg har rådført meg med radiolog

Det finnes klare retningslinjer

Stor risiko for falske positive/negative funn

Det er mitt ansvar som allmennlege i rollen som portvokter

Ønsker å vente å se hvordan tilstanden utvikler seg

Annet, spesifiser:

**Spesifiser her:**

*Dette elementet vises kun dersom alternativet «Annet, spesifiser:» er valgt i spørsmålet «Når du lar være å sende henvisninger til undersøkelser*

*du er usikker på om er nyttig for pasienten, hva er de vanligste årsakene til at henvisningen ikke sendes»*

**Bakgrunnsinformasjon**

**Er du godkjent spesialist i allmennmedisin?**

Ja

Nei

**I hvor mange år har du arbeidet innen allmennmedisin?**

Inkluderer både arbeid som LIS/assistentlege og spesialist.

<5 år

5-10år

>10år

**Hvordan er kapasiteten på bildediagnostiske tjenester i ditt område?**

Det er tilstrekkelig kapasitet i forhold til pasientgrunnlaget

Det er mangelfull kapasitet med lange ventetider for pasientene

**Hvor har du ditt daglige virke?**

Fastlegekontor

Legevakt

Privat helsetjeneste

Annet, spesifiser:

**Spesifiser her:**

*Dette elementet vises kun dersom alternativet «Annet, spesifiser:» er valgt i spørsmålet «Hvor har du ditt daglige virke?»*

*Generert: 2023-05-31 08:43:41.*
